# Breakpoint analysis of the recurrent constitutional t(8;22)(q24.13;q11.21) translocation

**DOI:** 10.1186/s13039-014-0055-x

**Published:** 2014-08-13

**Authors:** Divya Mishra, Takema Kato, Hidehito Inagaki, Tomoki Kosho, Keiko Wakui, Yasuhiro Kido, Satoru Sakazume, Mariko Taniguchi-Ikeda, Naoya Morisada, Kazumoto Iijima, Yoshimitsu Fukushima, Beverly S Emanuel, Hiroki Kurahashi

**Affiliations:** 1 Division of Molecular Genetics, Institute for Comprehensive Medical Science, Fujita Health University, Toyoake 470-1192, Aichi, Japan; 2 Department of Medical Genetics, Shinshu University School of Medicine, Matsumoto 390-8621, Nagano, Japan; 3 Department of Pediatrics, Dokkyo Medical University Koshigaya Hospital, Koshigaya 343-8555, Saitama, Japan; 4 Department of Pediatrics, Kobe University Graduate School of Medicine, Kobe 650-0017, Hyogo, Japan; 5 Division of Human Genetics, The Children’s Hospital of Philadelphia, Philadelphia 19104, PA, USA; 6 Department of Pediatrics, The Perelman School of Medicine of the University of Pennsylvania, Philadelphia 19104, PA, USA

**Keywords:** PATRR, t(8;22), Palindrome-mediated translocation, Supernumerary der(8)t(8;22)

## Abstract

**Backgrounds:**

The t(8;22)(q24.13;q11.2) has been identified as one of several recurrent constitutional translocations mediated by palindromic AT-rich repeats (PATRRs). Although the breakage on 22q11 utilizes the same PATRR as that of the more prevalent constitutional t(11;22)(q23;q11.2), the breakpoint region on 8q24 has not been elucidated in detail since the analysis of palindromic sequence is technically challenging.

**Results:**

In this study, the entire 8q24 breakpoint region has been resolved by next generation sequencing. Eight polymorphic alleles were identified and compared with the junction sequences of previous and two recently identified t(8;22) cases . All of the breakpoints were found to be within the PATRRs on chromosomes 8 and 22 (PATRR8 and PATRR22), but the locations were different among cases at the level of nucleotide resolution. The translocations were always found to arise on symmetric PATRR8 alleles with breakpoints at the center of symmetry. The translocation junction is often accompanied by symmetric deletions at the center of both PATRRs. Rejoining occurs with minimal homology between the translocation partners. Remarkably, comparison of der (8) to der(22) sequences shows identical breakpoint junctions between them, which likely represent products of two independent events on the basis of a classical model.

**Conclusions:**

Our data suggest the hypothesis that interactions between the two PATRRs prior to the translocation event might trigger illegitimate recombination resulting in the recurrent palindrome-mediated translocation.

## Background

The constitutional t(8;22)(q24.13;q11.2) is recognized as a one of several recurrent translocations in humans [1]. The most prevalent recurrent constitutional translocation is the t(11;22)(q23;q11) [2]. Although t(11;22) balanced-translocation carriers are phenotypically normal, they often manifest problems with reproduction such as infertility, recurrent pregnancy loss, or the birth of unbalanced offspring with the supernumerary der(22)t(11;22) syndrome (Emanuel syndrome [MIM 609029]) [3]. Among the small supernumerary marker chromosomes seen clinically, +der(22)t(11;22) is the most frequent, while + der(22)t(8;22) is the second most prevalent [4]. Similar to the t(11;22), balanced carriers of the t(8;22) are often identified after the birth of an unbalanced offspring with the supernumerary der(22) t(8;22), the phenotype of which includes extremity anomalies, mild dysmorphism and intellectual disability.

The mechanism that leads to the constitutional t(11;22)(q23;q11) has been extensively studied. The breakpoints of both chromosomes are consistently located within palindromic AT-rich repeats (PATRRs) [5]-[9]. Palindromic regions, i.e. inverted repeats, have a potential for the formation of hairpin/cruciform structures by intrastrand annealing and palindrome induced genomic instability has been demonstrated in many experimental model organisms [10]-[12]. In humans, a considerable number of *de novo* t(11;22)s arise during spermatogenesis, but *de novo* occurrences have not been detected in tissues other than sperm [13]. It has been proposed that the secondary structure of the palindromic DNA during spermatogenesis induces genomic instability leading to the recurrent chromosomal translocation [14],[15]. Taking advantage of breakpoint co-localization on 22q11, the translocation junction fragments of the t(8;22) have been isolated, the breakpoints on 8q24 were assessed, and a similar mechanism of translocation was suggested [1],[16]. Although PATRR-like sequence (PATRR8) was compiled from the junction sequences, detailed analysis of the breakpoint region have not been performed since the analysis of the palindromic region is technically challenging [17]. Further, since the database of human reference sequence does not include the complete sequence of PATRR8, details of the t(8;22) translocation mechanism are incomplete.

In this study, we first obtained the complete sequence of several polymorphic PATRR8 alleles from normal individuals using next generation sequencing. Using translocation-specific PCR, we also determined the translocation junctions in two unrelated Japanese families with the t(8;22)(q24.13;q11.2). We performed an investigation to examine the breakpoint within PATRR8 and PATRR22 by comparing the junction sequences with the normal PATRR8 and PATRR22. These data further confirm that the t(8;22) translocation is a recurrent rearrangement with a mechanism consistent with that proposed for the t(11;22) and the t(8;22) in previous studies. These findings provide additional support for the role of palindromic sequences in genomic instability. Further, our new finding, the similarity of the der(8) and the der(22) sequences, might elicit a new feature of palindrome-mediated translocations.

## Results

### Genomic structure of the PATRR8

Based on the putative PATRR8 sequences compiled by analysis of translocation junctions, the majority of PATRR8 is deleted and only a portion of the proximal arm appears in the human genome database [1]. To determine the complete sequence of PATRR8, we first attempted conventional PCR followed by standard Sanger sequencing. The sizes of the PCR products that include PATRR8 vary among individuals. We previously classified them into four categories: long (L), medium (M), short (S) and super-short (SS) [1]. The M and S alleles were the major alleles, while L and SS alleles were less frequent. Despite the fact that we could generate the complete sequence of the SS allele, their AT-rich and palindromic nature prevented us from sequencing the central region of the PATRR in other allele types [17].

**Figure 1:**
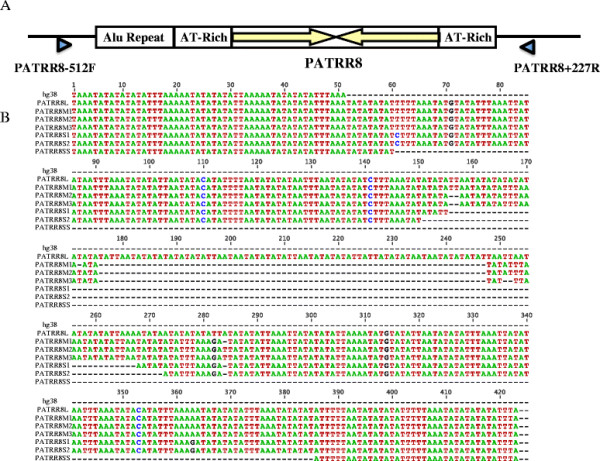
**Complete sequence of the polymorphic PATRR8 alleles. A**. Structure of PATRR8 with its flanking regions. Arrows indicate proximal and distal arms of the PATRR8. Arrowheads indicate PCR primers for amplification of PATRR8. **B**. Alignment of the sequences of PATRR8 polymorphic alleles.

Next, we attempted to sequence the PCR product by massively parallel sequencing using a next generation sequencer. Although the central region was under-represented (~50 reads out of ~30,000 reads per PCR product), we finally obtained the sequence of the entire PATRR8 in 11 out of 24 PCR products. Indeed, the sequence data obtained by next generation sequencing demonstrated that size polymorphisms of the PCR products result from size polymorphisms of PATRR8 itself as well as size variation in the flanking AT-rich repeat region (Figure 1A, Additional file 1: Figure S1).

The M allele (~350 bp), one of the most frequent variants, manifests a nearly perfect palindrome (Table 1). AT-richness is as high as 98%. Identity between the proximal and distal arms is >98%, showing a nearly perfect palindromic structure. Subtle nucleotide alterations produce three subtypes, M1, M2 and M3 (Figure 1B). The S allele (~310 bp), the other most frequent variant, also manifests a high AT-content (97%) and a perfect palindrome (identity 100%). The L allele (423 bp) and the SS allele (98 bp) are less frequent. The SS allele appears to be an asymmetrically deleted version of the S allele, whereas the L allele appears to have an asymmetric insertion of AT-rich sequence of unknown origin. The PATRR8 sequence appearing in the human genome database was not found to be a subtype of PATRR8 polymorphism. The deletion in the database carries a 16 bp homology at the junction (Additional file 1: Figure S1), suggesting that the sequence is an artifact generated during bacterial culture for clone preparation for sequencing.

**Table 1:** Characterization of the polymorphic PATRR8 alleles

**Allele**	**Size (bp)**	**AT content (%)**	%Identity*	**ΔG (kcal/mol)**	**Accession no**
PATRR8L	423	99%	92.7%	-142.71	AB968359
PATRR8M1	349	98%	99.4%	-139.20	AB968360
PATRR8M2	349	98%	98.3%	-132.12	AB968361
PATRR8M3	347	98%	98.3%	-131.36	AB968362
PATRR8S1	310	97%	100%	-125.90	AB968363
PATRR8S2	300	97%	100%	-122.22	AB968364
PATRR8SS	98	98%	96.0%	-33.22	AB969308

*%identity (similarity) between proximal and distal arms.

Unlike other translocation-related PATRRs, PATRR8 has another AT-rich flanking region both at its proximal and distal side. Both of these AT-rich regions manifest size polymorphisms. The proximal flanking region carries a 35 bp direct repeat, whose copy number is increased in M alleles (Additional file 1: Figure S1). The distal region also carries a similar 28 bp direct repeat, copy number variation of which produces size polymorphism. Since we could not distinguish between M and S alleles simply by gel electrophoresis due to these size polymorphisms in the flanking regions, we could not determine the exact frequency of polymorphic PATRR8 alleles in the general population.

### Analysis of the breakpoints of the der(8) and der(22)

**Figure 2:**
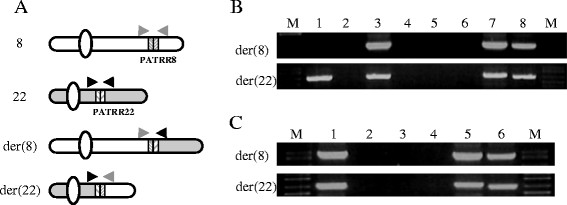
**The der(8) and the der(22) junction fragments of the t(8;22). A**. Diagram for the translocation-specific PCR system. Chromosome 8 is indicated in white, while chromosome 22 is depicted in grey. Hatched boxes indicate PATRR arms. Translocation-specific PCR was performed with one primer designed at the flanking region of PATRR8 (grey primers) and with the other primer at the flanking region of PATRR22 (black primers). **B**. Results of family 1. Upper panel indicates results for the der(8), while lower panel indicates those of the der(22). Lane M, size markers; lane 1, FHU13-031 (proband); lane 2, FHU13-032 (father); lane 3, FHU13-033 (mother); lanes 4 and 5, normal healthy controls; lane 6, water control; lanes 7 and 8, balanced t(8;22) translocation carriers unrelated to the family. **C**. Results of family 2. Lane M, size markers; lane 1, FHU13-027 (proband); lanes 2 and 3, normal healthy controls; lane 4, water control; lanes 5 and 6, balanced t(8;22) translocation carriers unrelated to the family.

Using primers flanking PATRR8 and PATRR22 (Figure 2A), genomic DNAs from all of the t(8;22) cases yielded translocation specific PCR products (Figure 2B). Approximately 850 bp of the der(8) and 650 bp of the der(22) harboring the translocation junction were amplified by PCR from balanced translocation carriers in family 1 (FHU13-033) and family 2 (FHU13-027) as well as from the unrelated balanced translocation carriers published previously [1]. Only the der(22) PCR product was amplified from the proband in family 1 (FHU13-031) with the typical supernumerary der(22)t(8;22). Now that we have the complete sequence of PATRR8, we can compare the junction sequences with the putative original sequences. Based on sequence polymorphisms at the center and on the arm regions of PATRR8, we can deduce the original allele types. We suggest that FHU13-033 (family 1) as well as case 13 originated from PATRR8M, while FHU13-027 (family 2) as well as cases 8, 9, 12 and 16 originated from PATRR8S1 (Table 2). Regarding the PATRR22, FHU13-033 (family 1) and FHU13-027 (family 2) originated from PATRR22C, while cases 12 and 13 originated from PATRR22A. PATRR22 sequence in case 16 was so diverged from known PATRR22 variants that we could not determine the origin. Virtually all of the translocations occurred on symmetrical alleles of PATRR8 and PATRR22.

**Table 2:** Origin of the PATRR subtypes

**Sample name**	**PATRR8**	**PATRR22**	**Reference**
Family 1(FHU13-033)	PATRR8M	PATRR22C	This study
Family 2 (FHU13-027)	PATRR8S1	PATRR22C	This study
Case 8*	PATRR8S1	ND**	Sheridan et al. 2010 [1]
Case 9*	PATRR8S1	ND**	Sheridan et al. 2010 [1]
Case 12 (CH00-180)	PATRR8S1	PATRR22A	This study (Sheridan et al. 2010) [1]
Case 13 (CH07-194)	PATRR8M	PATRR22A	This study (Sheridan et al. 2010) [1]
Case 16	PATRR8S1	NA***	Sheridan et al. 2010 [1]

*Only the der(22) was analyzed.

**Not determined, ***Not applicable.

**Figure 3:**
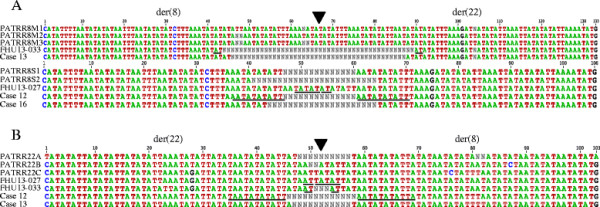
**Sequence comparison between the der(8) and the der(22) junction fragments with the putative original PATRRs. A**. Compilation of the chromosome 8 side of the der(8) and der(22) with the PATRR8. **B**. Compilation of the chromosome 22 side of the der(8) and der(22) with the PATRR22. Triangles indicate the center of the PATRRs. Nucleotides participating in microhomology are underlined.

When the chromosome 8 portions of the der(8) and der(22) were aligned with PATRR8, the central region often appeared to be deleted (Figure 3A). Although the extent of deletion differs amongst cases, the sequence derived from the proximal and the distal arms shows loss of the same number of nucleotides from PATRR8. Likewise, the deletion is symmetrically located at the center of PATRR22 (Figure 3B). This suggests that the breakage always occurred at the center of the palindrome followed by a progression of bidirectional deletion.

### Analyses of the junctions of the der(8) and der(22)

We analyzed the junctions of PATRR8 and PATRR22 both for the der(8) and der(22). No substantial homology could be observed between PATRR8 and PATRR22 (35-50% similarity). We only observed a few identical nucleotides at the point where the original PATRR8 and PATRR22 sequences were joined (2-11 bp) (Figure 3A, B). Both PATRR8 and PATRR22 are so highly AT-rich that even homology-independent rejoining could manifest some microhomology at the junction by chance [9]. Therefore, the molecular pathways that are assumed to drive generation of this translocation might include microhomology-mediated end joining, classical non-homologous end joining, or alternative non-homologous end joining.

### Comparison between the der(8) and der(22) sequences

**Figure 4:**
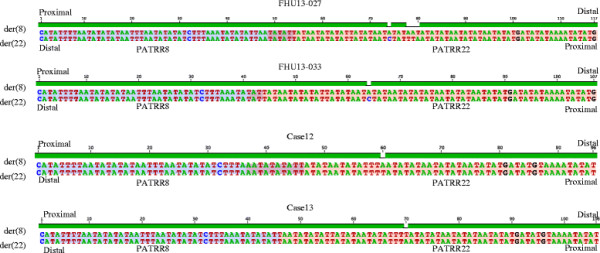
**Sequence comparison between der(8) and the der(22) junction fragments in each case.** Sequences are shown from PATRR8 side (blue) to the PATRR22 side (pink). Nucleotides participating in microhomology are shown in purple.

We further compared the junction sequences of the der(8) and the der(22). Strikingly, the der(8) and the der(22) sequences were identical at the junctions in all cases (Figure 4), although subtle nucleotide differences were identified in the arm region that reflected nucleotide differences between the proximal and distal arms. On the basis of a standard mechanism of translocation formation based on double-strand DNA repair, formation of the der(8) and the der(22) occur independently [18]. If there were a long stretch of homologous sequence at the junction, there would be a chance to produce the same junction fragments independently. However, even at the junction with microhomology of only a few nucleotides, the der(8) and the der(22) sequences were identical.

## Discussion

### Characterization of the PATRR8

In this study, we determined the entire PATRR8 sequence in humans for the first time. All of the translocation-related PATRRs identified to date have three common features. 1) They comprise nearly perfect palindromes, whose lengths are several hundred base pairs. 2) An AT-rich region is located at the center, while there are relatively non-AT-rich regions at both ends. 3) Another AT-rich region exists flanking the PATRR [9]. Although non-AT-rich regions were absent within PATRR8, it possesses all three common features of translocation related PATRRs. It is proposed that all of these features foster the propensity for forming secondary structure at palindromic regions. Indeed, PATRR8 contributes to generation of not only the t(8;22), but also the constitutional t(3;8) that is associated with hereditary renal cell carcinoma predisposition, suggesting that the PATRR8 is a hotspot for palindrome-mediated translocations [19]. It is likely that for the t(8;22), as for other PATRR-related recurrent translocations such as the t(11;22) and t(17;22), DNA secondary structure might contribute to the generation of the translocation.

Similar to other translocation-related PATRRs, PATRR8 manifests size polymorphisms due to those within the PATRR itself as well as those in the flanking AT-rich region. Although minor variations are present at the nucleotide level, size variations of PATRR8 were found to be of only four types; two symmetric types and two asymmetric types. This might imply that PATRR8 is generally transmitted stably and is not predisposed to insertion or deletion. Alternatively, it is possible that PATRR8 might have emerged recently during evolution. Similar to other recurrent PATRR-mediated translocations, the t(8;22) was found to arise from symmetric variants [20],[21]. This indirectly but strongly suggests that PATRR8 adopts secondary structures *in vivo*.

### Clinical significance for translocation-specific PCR

In all cases, both translocation breakpoints are located within several hundred base pair intervals on each chromosome, which could be identified with primers flanking PATRR8 and PATRR22. Similar to the t(11;22), this translocation-specific PCR is diagnostic since it can detect all of the t(8;22) translocations [22]. For example, in examination of a case like FHU13-027 with a balanced t(8;22) with intellectual disability and mild dysmorphic features, it might be difficult to know if the t(8;22) translocation is responsible for the phenotype as a result of breakpoint variation. On the basis of positive t(8;22)-specific PCR for both derivatives, we could conclude that the case is a standard t(8;22) balanced carrier and the t(8;22) translocation itself was unlikely to be the cause of the phenotype. Such translocation-specific PCR can also be useful in determining the origin of a small supernumerary marker chromosome of unknown origin. Since the t(8;22) is the second most frequent amongst small supernumerary marker chromosomes [4], t(8;22)-specific PCR is a simple and cost-effective method for marker identification as compared to multicolor spectral karyotyping for example.

Among the conceptions with unbalanced translocation products from a balanced t(8;22) translocation carrier that might result in early pregnancy loss, only a fetus with + der(22) karyotype through meiotic 3:1 segregation might be viable. Prenatal diagnosis for supernumerary der(22)t(8;22) syndrome could be performed via chorionic villus biopsy or amniocentesis. Non-invasive prenatal testing might also be possible, particularly if the male partner is a balanced translocation carrier. Further, translocation-specific PCR can also be applied for pre-implantation diagnosis using DNA amplified by whole genome amplification methods using the genomic DNA from a blastomere or blastocyst biopsy.

### Possible mechanism for palindrome-mediated translocation

PATRR-mediated genomic instability is likely to occur via two distinct mechanisms; replication-dependent and replication-independent [23],[24]. The replication-dependent route is induced by replication fork stalling as a result of a hairpin structure within the lagging-strand template during DNA replication [25]. This is followed by template switching via microhomology leading to gross chromosomal rearrangements like translocations [26] Indeed, this kind of somatic rearrangement is often identified in cancer cells [27]. However, translocation-specific PCR only detects the t(8;22) as well as the t(11;22) in sperm, suggesting that PATRR-mediated translocations arise in gametogenesis, most notably spermatogenesis [13],[28]. One of the explanations for spermatogenesis-specific palindrome-mediated genomic instability is that during spermatogenesis a significant number of DNA replications take place. This would be a pre-meiosis hypothesis [2],[29]. Indeed, PATRR17 located within an intron of the NF1 gene contributes to some germ-line gross chromosomal rearrangements such as deletions and translocations resulting in neurofibromatosis type 1 [30]. The breakpoint features of these rearrangements are distinct from PATRR-mediated translocations [31],[32].

An alternative hypothesis is a post-meiosis hypothesis, which is based on replication-independent cruciform structure formation at the PATRRs by free negative supercoiling induced by extensive histone removal during late spermatogenesis. Symmetrical deletions on both the proximal and distal arms might imply that the deletions do not occur after DNA breakage at the central region of the PATRR followed by dissociation of the proximal and distal arms. Perhaps they occur after the central breakage with the PATRR maintaining its secondary structure, upon annealing of the proximal and distal arms. The symmetrical deletions are reflected in the identical nature of the der(8) and the der(22) sequences, which must be generated as independent events based on a classical DSB repair model for translocation formation [18]. The identical sequence of the der(8) and the der(22) might imply that rejoining occurs between the PATRRs while still keeping their secondary structure. Finally, the partner chromosome of a PATRR-mediated translocation is always another PATRR [2]. Thus, the hairpin-hairpin model proposed by Inagaki et al. might represent a plausible model for PATRR-mediated translocations in humans [33].

## Conclusions

In our current study, comparison of der(8) to der(22) sequences shows identical breakpoint junctions between them, which likely represent products of two independent events on the basis of a classical model. Our data suggest the hypothesis that interactions between the two PATRRs prior to the translocation event might trigger illegitimate recombination resulting in the recurrent palindrome-mediated translocation.

## Methods

### Human samples

**Figure 5:**
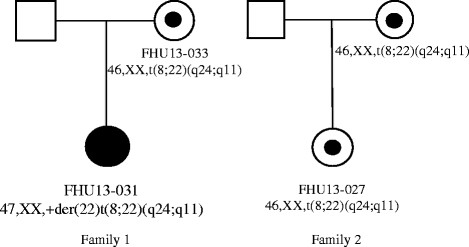
Family pedigrees for two newly identified t(8;22) families.

In this study, we used genomic DNA samples from cases 12 (CH00-180) and 13 (CH07-194) from the previous study [1]. In addition, we identified two new families of Japanese origin with the t(8;22)(q24;q11) translocation (Figure 5). One family (family 1) was identified through a female proband (FHU13-031) with typical features of the supernumerary der(22)t(8;22) syndrome characterized by clinodactyly, mild dysmorphia with preauricular pit, and intellectual disability. Her normal healthy mother was a balanced t(8;22) translocation carrier (FHU13-033). The other family (family 2) was identified by a female proband who was a balanced t(8;22) translocation carrier (FHU13-027) revealed by screening based upon intellectual disability and mild dysmorphia. The normal healthy mother also carried the same translocation. After informed consent was obtained, peripheral blood samples were obtained. This study was approved by the Ethical Review Board for Human Genome Studies at Fujita Health University (Accession number 145, approved on 16 April 2013).

### Next generation sequencing

Genomic DNA was purified by QuickGene-610 L (Fuji Film). PATRR8 was amplified with primers flanking PATRR. PATRR8-512 F (5′-GATTACATATGGCATCTGGTAGGCTG-3′) was used as the forward primer and PATRR8 + 227R (5′-GTGCCAAAATGTCAAGTCATCTGTG-3′) was used as the reverse primer. PCR was performed with the KAPA Extra (KAPA Biosystems). The PCR products were separated by 2% agarose gel electrophoresis and the genotypes for size polymorphism were determined.

To obtain the entire PATRR8 sequence, we used five t(8;22) balanced translocation carriers, who carry only one copy of the intact PATRR8. In addition, we selected 19 normal healthy donors who were heterozygous for size polymorphisms of PATRR8. The PCR products were separated by 2% agarose gel electrophoresis and each PCR product derived from a different allele was purified separately.

For next generation sequencing, tagmentation were performed using a Nextera XT DNA sample prep kit (Illumina) according to the manufacturer’s specifications. The libraries were amplified using the KAPA Library Amplification Kit (KAPA Biosystems) with the Nextera Index Kit to add indices and common adapters for subsequent cluster generation and sequencing. Prior to cluster generation, normalized libraries were further quantified by Qubit (Invitrogen Q32866) using the Qubit dsDNA HS Assay Kit (Invitrogen Q32851) and the 2100 Bioanalyzer (Agilent Technologies) using the High Sensitivity DNA Kit (Agilent Technologies, 5067–4626). PhiX control was added to the reaction to increase sequence diversity. Finally, the prepared library was loaded on an Illumina MiSeq clamshell style cartridge for paired end sequencing (Illumina). The data were analyzed using CLC Genomics Workbench. After trimming, reads were assembled as *de novo* assemblies or they were mapped to putative references prepared from junction fragments derived from t(8:22) translocation carriers to produce consensus sequences. Identity was calculated by Emboss Needle software, while ΔG was calculated by mfold.

### PCR amplification of the junction fragments

To amplify an ~850 bp product containing the der(8) breakpoint junction fragment and to amplify the ~650 bp product containing the der(22) breakpoint junction fragment, a two-step PCR system was used [17]. The der(8) products were amplified with PATRR8-512 F and PATRR22 + 178R (5′-CATGATTCTGGATAACTTCCAAA-3′) or JF22 (5′-CCTCCAACGGATCCATACT-3′) primers, while the der(22) products were amplified with PATRR8 + 227R and PATRR22-394 F (5′-TCAGTTTATTCCCAAACTCCCAAAT-3′) or JF22 primers. PCRs were performed using LA Taq DNA Polymerase (Takara) and the PCR conditions were as follows: 94°C for 2 min, followed by 35 cycles of 98°C for 30 s, 60°C for 5 min. The resulting PCR products were checked on 2% agarose gels, subjected to ExoSAP-IT digestion (Affymetrix), and then sequenced bidirectionally by capillary electrophoresis (ABI3730 Genetic Analyzer, Applied Biosytems). Sequences were analyzed with Clustalw2, which was used to align the resulting sequences.

## Competing interests

The authors declare that they have no competing interests.

## Authors’ contributions

DM - Participated in the design of the study, carried out the molecular biology work, and drafted the manuscript. TKA - Participated in the design of the study, carried out the molecular biology work, and drafted the manuscript. HI - Participated in the design of the study, carried out the molecular biology work. TKO - Participated in the design of the study, carried out the molecular biology work. KW - Participated in the design of the study, carried out the molecular biology work. YK - Participated in the design of the study, carried out the molecular biology work. SS - Participated in the design of the study, carried out the molecular biology work. MTI - Participated in the design of the study, carried out the molecular biology work. NM - Participated in the design of the study, carried out the molecular biology work. KI - Participated in the design of the study, carried out the molecular biology work. YF - Participated in the design of the study, carried out the molecular biology work. BSE - Coordinated and conceived the study, being involved in the critical revision of the manuscript for important intellectual content. HK - Coordinated and conceived the study, participated in the design of the study, drafted the manuscript, being involved in the critical revision of the manuscript for important intellectual content. All authors have read and approved the final manuscript.

## Additional file

## Supplementary Material

Additional file 1: Figure S1. Complete sequence of the polymorphic PATRR8 with flanking regions. Large arrows indicate the proximal and distal PATRR arms. Direct repeats at the flanking regions proximal and distal to the PATRR8 are underlined (blue solid or dotted lines). The black lines indicate homology between the proximal and distal region to the PATRR8 deletion that appears in the human genome database. Click here for file
